# Complete Genomic Sequences of Three Salmonella enterica subsp. *enterica* Serovar Muenchen Strains from an Orchard in San Joaquin County, California

**DOI:** 10.1128/MRA.00048-20

**Published:** 2020-03-26

**Authors:** Thao D. Tran, Steven Huynh, Craig T. Parker, Robert Hnasko, Lisa Gorski, Sat Darshan S. Khalsa, Patrick H. Brown, Jeffery A. McGarvey

**Affiliations:** a Department of Plant Sciences, University of California, Davis, California, USA; b Produce Safety and Microbiology Research Unit, USDA, ARS, Albany, California, USA; c Foodborne Toxin Detection and Prevention Research Unit, USDA, ARS, Albany, California, USA; Indiana University, Bloomington

## Abstract

We present here the complete genome sequences of three Salmonella enterica subsp. *enterica* serovar Muenchen strains, LG24, LG25, and LG26. All three strains were isolated from almond drupes grown in an orchard in San Joaquin County, California, in 2016. These genomic sequences are nonidentical and will contribute to our understanding of S. enterica genomics.

## ANNOUNCEMENT

Salmonella enterica subsp. *enterica* serovar Muenchen (*Salmonella* Muenchen) is one of 2,449 serotypes of the genus *Salmonella* (1, 2). It accounted for 6,055 of the 441,863 (1.3%) documented cases of *Salmonella* infection in the United States between 1987 and 1997 and has been linked to several multistate foodborne outbreaks (1, 3). The three *Salmonella* Muenchen strains (LG24, LG25, and LG26) in this study were isolated from almond drupes grown in an orchard located in San Joaquin County, California, in 2016. Almond drupes were picked from almond trees with a gloved hand, placed in sterile plastic bags, placed on ice, and sent to the USDA, Western Regional Research Center, within 8 h. The drupes were assayed for Salmonella enterica by the immunomagnetic separation (IMS) method described by Gorski et al. in 2011 (4). Briefly, samples were enriched in tryptic soy broth (TSB) at 37°C for 16 h. A 100-μl aliquot was plated on modified semisolid Rappaport-Vassiliadis (MSRV) agar, and a second 0.5-ml aliquot was mixed with Dynal anti-*Salmonella* beads (Invitrogen, Carlsbad, CA) in a 1.5-ml tube and processed with a Dynal BeadRetriver (Invitrogen) as described by the manufacturer. The processed beads were suspended in 3 ml of Rappaport-Vassiliadis soya peptone (RVS) broth (Oxoid, Cambridge, UK) and incubated at 42°C for 16 h. Broth samples with obvious growth were plated onto xylose lysine deoxycholate (XLD) agar; black colonies were selected and confirmed to be Salmonella enterica by 16S rRNA gene analysis as described.

*Salmonella* Muenchen strains LG24, LG25, and LG26 were grown aerobically on tryptic soy agar (TSA) for 24 h at 37°C. For DNA extraction, a single colony was cultured overnight in 100 ml of tryptic soy broth (TSB) (Oxoid, Basingstoke, Hampshire, England) for 24 h at 37°C with shaking at 200 RPM. Genomic DNA was extracted by the sucrose-Tris with phenol-chloroform cleanup method as described previously (5).

Sequencing was carried out using the PacBio RS II (Pacific Biosciences, Menlo Park, CA) and Illumina MiSeq platforms. PacBio and Illumina MiSeq sequencing were performed using standard protocols, with libraries constructed as described (6). For the PacBio platform, SMRTbell libraries were prepared from 10 μg of bacterial genomic DNA with G-TUBE (Covaris, Woburn, MA) fragmentation following a modification of the PacBio procedure (7) using 1× AMPure cleanup and DNA repair after 10-kb size selection using BluePippin with a 0.75% DF Marker S1 high-pass 6- to 10-kb vs3 cassette (Sage Science, Beverly, MA). Single-molecule real-time (SMRT) cells were run with 0.1 nM on-plate concentration, P6/C4 sequencing chemistry, the MagBead OneCellPerWell v1 collection protocol, and 360-min data collection mode. For the Illumina platform, libraries were prepared with the KAPA LTP library preparation kit (Kapa Biosystems, Wilmington, MA). The pooled libraries were loaded into a MiSeq system and sequenced using a MiSeq reagent kit v2 with 2 × 250 cycles (Illumina, Inc.). The PacBio reads were assembled using the RS Hierarchical Genome Assembly Process (HGAP) v3.0 in SMRT Analysis v2.2.0 (Pacific Biosciences). A final base call validation of the PacBio contigs was performed by mapping Illumina MiSeq reads, trimmed using a quality score threshold of 30 or higher (Q30), to the PacBio assembly and the reference assembler within Geneious Prime (2019.2.3) (Biomatters, Ltd., Auckland, New Zealand). Single nucleotide polymorphisms (SNPs) between the PacBio assembly and the MiSeq reads were addressed using the annotate and predict/find SNPs module, with a minimum coverage parameter of 50 and a minimum variant frequency parameter of 0.8. PacBio DNA internal control complex P6 was used as an internal sequencing control, and the read quality control was conducted using FastQC (PacBio). Protein-, rRNA-, and tRNA-coding genes were annotated using the NCBI Prokaryotic Genome Automatic Annotation Pipeline (PGAAP) (8), with additional manual annotation based on the genome of Salmonella enterica subsp. e*nterica* strain RM11060 (GenBank accession number CP022658.1) (9).

For strain LG24, the PacBio RS II platform produced 40,548 total reads, and 38,371 reads were used for assembly (*N*_50_, 17,234 bp). The Illumina MiSeq platform yielded 2,159,434 total reads, and 2,041,230 reads were used for assembly. For strain LG25, the PacBio RS II platform produced 56,094 total reads, and 53,059 reads were used for assembly (*N*_50_, 16,650 bp). The Illumina MiSeq platform yielded 1,949,310 total reads, and 1,839,428 reads were used for assembly. For strain LG26, the PacBio RS II platform produced 63,205 total reads, and 58,418 reads were used for assembly (*N*_50_, 19,914 bp). The Illumina MiSeq platform yielded 1,713,524 total reads, and 1,617,507 reads were used for assembly. The PacBio assembly produced 4 contigs for strains LG24 and LG26 and 3 contigs for strain LG25. The accession numbers and the assembly metrics for each complete genome are listed in Table 1.

**TABLE 1:** Characteristics and accession numbers of genomes and plasmids of *Salmonella* Muenchen strains

*Salmonella* Muenchen strain	Size (bp)	Coverage (×)^a^	No. of plasmids	No. of CDSs^b^	G+C content (%)	No. of rRNA operons	No. of tRNAs	No. of prophage regions (intact/questionable/incomplete)	No. of ISs^c^	GenBank accession no.
LG24										
LG24Chr	4,930,424	161.0	3	5,138	52.2	7	85	4/2/1	207	CP045056
pLG24p1	26,060	161.0						0/0/0		CP045053
pLG24p2	270,881	161.0						1/1/0		CP045054
pLG24p3	40,386	161.0						0/0/0		CP045055
LG25										
LG25Chr	4,930,422	172.0	2	5,085	52.2	7	85	4/2/1	207	CP045059
pLG25p1	26,057	172.0						0/0/0		CP045057
pLG25p2	270,878	172.0						1/1/0		CP045058
LG26										
LG26Chr	4,930,420	179.0	3	5,142	52.2	7	85	4/2/1	207	CP045063
pLG26p1	26,056	179.0						0/0/0		CP045060
pLG26p2	270,863	179.0						1/1/0		CP045061
pLG26p3	40,385	179.0						0/0/0		CP045062

a Coverage was combined from PacBio and Illumina platforms.

b CDSs, coding sequences; prophage regions were accessed by PHASTER (10) in November 2019.

c ISs, insertion sequences; ISs were determined with ISfinder (11).

### Data availability.

The whole-genome sequence has been deposited in DDBJ/ENA/GenBank under the accession numbers CP045056 (LG24 chromosome), CP045053 (pLG24p1), CP045054 (pLG24p2), and CP045055 (pLG24p3) (BioProject, PRJNA576703; BioSample, SAMN13002763); CP045059 (LG25 chromosome), CP045057 (pLG25p1), and CP045058 (pLG25p2) (BioProject, PRJNA576705; BioSample, SAMN13002952); and CP045063 (LG26 chromosome), CP045060 (pLG26p1), CP045061 (pLG26p2), and CP045062 (pLG26p3) (BioProject, PRJNA576706; BioSample, SAMN13002973). The raw PacBio data are available via SRA under the accession numbers SRR10283793 (LG24), SRR10284761 (LG25), and SRR10286355 (LG26). The raw Illumina data are available via SRA under the accession numbers SRR11101341 (LG24), SRR11101395 (LG25), and SRR11101397 (LG26).
